# Boosting the Antioxidant Potential of Polymeric Proanthocyanidins in Litchi (*Litchi chinensis* Sonn.) Pericarp via Biotransformation of Utilizing *Lactobacillus* Plantarum

**DOI:** 10.3390/foods12122384

**Published:** 2023-06-15

**Authors:** Haocheng Liu, Yuqian Tang, Zhaowen Deng, Jiguo Yang, Dan Gan

**Affiliations:** 1 School of Food Science and Engineering, South China University of Technology, Guangzhou 510641, China; ansishc@163.com (H.L.); tangsiuman@outlook.com (Z.D.); 2 Sericultural & Argi-Food Research Institute, Guangdong Academy of Agricultural Sciences/Key Laboratory of Functional Foods, Ministry of Agriculture and Rural Affairs/Guangdong Key Laboratory of Agricultural Products Processing, No.133 Yiheng Street., Dongguanzhuang Road, Tianhe District, Guangzhou 510610, China; 3 Heyuan Branch, Guangdong Laboratory for Lingnan Modern Agriculture, Heyuan 517000, China; yqtang@scut.edu.cn; 4 Sirio Pharma Co., Ltd., Shantou 515000, China

**Keywords:** litchi pericarp, polymeric proanthocyanidins, biotransformation, antioxidant activity

## Abstract

In order to enhance the efficient utilization of polymeric proanthocyanidins from litchi pericarp, a process for transforming litchis’ polymeric proanthocyanidins (LPPCs) by using *Lactobacilli* has been established for products with highly antioxidative properties. *Lactobacillus plantarum* was selected to enhance the transformation effect. The transformation rate of LPPCs reached 78.36%. The content of litchis’ oligomeric proanthocyanidins (LOPCs) in the products achieved 302.84 μg grape seed proanthocyanidins (GPS)/mg DW, while that of total phenols was 1077.93 gallic acid equivalents (GAE) μg/mg DW. Seven kinds of substances have been identified in the products by using the HPLC-QTOF-MS/MS method, among which 4-hydroxycinnamic acid, 3,4-dihydroxy-cinnamic acid, and proanthocyanidin A2 were major components. The in vitro antioxidative activity of the products after transformation was significantly (*p* < 0.05) higher than those of LOPCs and LPPCs. The scavenging activity of the transformed products for DPPH free radicals was 1.71 times that of LOPCs. The rate of inhibiting conjugated diene hydroperoxides (CD-POV) was 2.0 times that of LPPCs. The scavenging activity of the products for ABTS free radicals was 11.5 times that of LPPCs. The ORAC value of the products was 4.13 times that of LPPCs. In general, this study realizes the transformation of polymeric proanthocyanidins into high-activity small-molecule substances.

## 1. Introduction

Litchi (*Litchi chinensis* Sonn.) originates from the southern part of China and is known as the “King of Fruits in Lingnan” for white jade-like transparent flesh, plumpness, and succulency. It is very popular among consumers and is an important tropical and subtropical fruit in China [1,2]. In 2022, China’s litchi production reached 2.531 million tons. Due to a continuous expansion of litchi production, the demand for eating fresh litchis has been far lower than the production capacity of litchis [3]. The development of the litchi processing industry leads to abundant by-products, among which litchi pericarp accounts for 15–20% of the fresh weight of a single litchi fruit. The pericarp is inedible and only a small amount of pericarp will be made into Chinese herbal medicines [1,4]. A large amount of pericarp will be discarded, resulting in a waste of resources and environmental pollution.

Litchi pericarp has abundant active substances such as phenolic acids, flavonoids, and polysaccharides, and contains rich proanthocyanidins (PC). Proanthocyanidins, a class of plant polyphenols widely existing in nature, have various biological activities such as inhibiting inflammation and cancers, delaying aging and controlling body weight [5,6]. However, a large number of studies indicate that the bioavailability of proanthocyanidins is greatly affected by the degree of polymerization. Polymeric proanthocyanidins, after their entry into a human body, will be directly excreted and, thus, fail to be absorbed [7,8]. Our previous study showed that the content of proanthocyanidins in litchi pericarp (LPCs) reached 7.54%, while the acquisition rate of LPPCs was 3.40%. LPPCs made up 45.21% of the total proanthocyanidins, containing nearly half of high polymers. To achieve a comprehensive application of proanthocyanidins, recycling high polymers and further transforming them into oligomers or small-molecule active substances is an effective way for efficient utilization of by-products from litchi pericarp.

At present, the relatively mature methods for transforming high polymers into oligomers include chemical methods such as acid hydrolysis, alkaline hydrolysis, rare metal-catalyzed hydrogenolysis, etc. However, these methods have many disadvantages including a large loss of active ingredients, long cycles, complex processes, and a low extraction rate [9,10]. In terms of the biological method, proanthocyanidins are transformed into non-proanthocyanidin products with higher activity and higher bioavailability via microbial metabolism [11,12]. Related studies reveal that polymeric proanthocyanidins can be metabolized and degraded by intestinal microorganisms in the process of intestinal accumulation, resulting in the production of active small-molecule substances such as hydroxyphenyl-γ-valerolactone and phenolic acids, which will be reabsorbed by the intestinal tract and enter the circulatory system [13,14]. Hence, the positive effect of proanthocyanidins on human health comes from not only the absorbed part but also the part that is transformed into small-molecule active substances after degradation of microorganisms. The microorganisms used in existing studies generally include lactic acid bacteria and mold. *Lactobacilli*, a genus of the representative probiotics and one indispensable important group of bacteria for human bodies, are usually distributed in the gastrointestinal tract and play an active role in keeping human health [15,16]. In recent years, there have been more studies on the transformation of substances rich in phenols by applying probiotics. After some effects from the catalytic activity of enzymes, such as deglycosylation, ring fission, dehydroxylation, etc., the substances rich in phenols are transformed into the products with higher bioavailability and bioactivity [14,17]. However, the transformation of proanthocyanidins by using *Lactobacilli*, with monomers and oligomers as substrates has not been completely explored and developed, and there are few reports on the transformation of proanthocyanidins by using high polymers as substrates [18,19].

This study aims to (1) select proper transformation-purpose strains from five common *Lactobacillus* species according to their growth and metabolism conditions, transformation rates of LPPCs, and the contents of (oligomeric) proanthocyanidins and total phenols in the products from transformation, and (2) analyze the products by using the HPLC-QTOF-MS/MS technique to observe the changes in the antioxidative activity of LPPCs before and after transformation, further finding a way to transform low-value raw materials into high-value active products and facilitating the comprehensive application of litchi pericarp and proanthocyanidins.

## 2. Materials and Methods

### 2.1. Materials and Reagents

The raw material used in this study was Litchi chinensis Sonn.cv. Nuomici, coming from the litchi orchard in Conghua District, Guangzhou (113°55′ E, 23°57 N). After cleaning, the litchi pericarp was collected, then frozen in liquid nitrogen and freeze-dried (−80 °C for 72 h; Coolsafe Benchtop, Scanvac, Sweeden). The freeze-dried litchi pericarp was crushed and screened by using an 80-mesh sieve. The litchi pericarp powder was stored in a −20 °C refrigerator in the dark for later use. The strains for experiments came from the China General Microbiological Culture Collection Center, including *Lactobacillus fermentum* (GIM 1.731), *Lactobacillus acidophilus* (GIM 1.204), *Lactobacillus plantarum* (GIM 1.648), *Lactobacillus casei* (BNCC 195633), and *Lactobacillus paracasei* (GIM 1.985). 4-(Dimethylamino) cinnamaldehyde, proanthocyanin, dingallic acid, and (HPLC ≥ 98%) standard substances were purchased from Shanghai Yuanye Biotechnology Co., Ltd. Reagents consisting of nutrient agar, yeast extract, beef extract, triammonium citrate, potassium dihydrogen phosphate, ethanol, and ethyl acetate, were purchased from TCI Co. Ltd. (Shanghai, China). Ultrapure water was prepared by a Milli-Q system purchased from Millipore (Billerica, MA, USA).

### 2.2. Preparation of LPPCs and LOPCs from Litchi Pericarp

Based on the previous research achievements of our team, LPCs in litchi pericarp were extracted by using the ultrasonic-microwave synergistic extraction (UMSE) method, the optimal process parameters were a microwave power 307 W, extraction time 17 min, extraction temperature 46 °C, and material–liquid ratio 1:31 g/mL. LPCs were used as raw materials for the separate preparation of LOPCs and LPPCs. Specific processes were described as follows. Firstly, LPCs aqueous solution was extracted for degreasing treatment by the *n*-hexane with a volume three times that of LPCs. Then, AB-8 macroporous resin was used for preliminary purification of LPCs aqueous solution, and ethyl acetate was utilized for extraction. After the removal of ethyl acetate by distillation under reduced pressure, and freeze-drying, LOPCs were obtained. Sephadex LH-20 column chromatography was adopted for separation of the remaining aqueous phase, removing other phenolic impurities such as hydrolysable tannins and anthocyanins. Later, the eluate was collected. After the removal of acetone by distillation under reduced pressure, the aqueous solution was collected. After freeze-drying, LPPCs were acquired, sealed and stored at 20 °C in the dark for further use [20,21].

### 2.3. Screening of Strains for Biotransformation of LPPCs from Litchi Pericarp

#### 2.3.1. Activation of Strains

The powder of five species of *Lactobacillus* was dissolved in sterile water. The mixture was inoculated on MRS solid medium after diluting to a certain extent. After sealing, it was cultured at 37 °C for 48 h. The streak plate method was adopted for selecting a single bacterial colony with a good shape. Three serial subcultivations were carried out. The whole process was performed aseptically to avoid contamination.

#### 2.3.2. Biotransformation Process

A single active bacterial colony of each different strain was inoculated on MRS liquid medium, respectively, and cultured at 37 °C for 16 h. On a basis of the previous experiments, a solution of activated bacteria of each strain was added to the MRS liquid medium containing LPPCs of 0.15 mg/mL at an inoculum size of 1% (added inoculum concentration reached about 10^7^ CFU/mL), which was then fermented at 37 °C for 72 h after sealing. The optical density (OD)_600nm_ values of the fermentation broth were measured at 0, 2, 4, 6, 8, 10, 12, 24, 36, 48, 60, and 72 h. The culture time and absorbance value were taken as the horizontal and vertical coordinates, respectively, to produce a growth curve for each strain, with the growth curves of *Lactobacillus* without adding LPPCs as a control. The pH values at 0, 24, 48, and 72 h were measured, with the pH values of *Lactobacillus* without adding LPPCs at various time points as a control to study the growth and metabolism of each strain. The MRS culture solution containing LPPCs of 0.15 mg/mL without inoculation of bacteria solutions was adopted as a control group to exclude the interference from natural degradation of LPPCs. All inoculation and sampling operations were performed on a clean bench.

#### 2.3.3. Preparation of Products from Transformation and Determination of Transformation Rate of LPPCs

After the fermentation, the fermentation broth was ultrasonically processed in an ice bath for 20 min to facilitate the rupture of bacteria for releasing the contents. Centrifugation was performed at 5000 rpm for 15 min to separate the bacteria. The supernatant fermentation broth was extracted with *n*-hexane. Degreasing processing was conducted for removing non-polar substances such as lipids. Operations were repeated three times. Then, the ethyl acetate with a volume three times that of the remaining substances was used for extraction to obtain the products. This operation was repeated five times. After removing the ethyl acetate by distillation under reduced pressure, methanol was adopted for redissolution. The remaining products were placed in a refrigerator (−20 °C) for measurement [20].

After the preparation of products according to the above procedures, the remaining fermentation broth was freeze-dried. Then, the untransformed LPPCs were recovered and extracted by using the extraction process in Section 2.2. The content of proanthocyanidins in the extract was measured by applying method 1 in Supplementary Materials to calculate the transformation rate of LPPCs.

Transformation rate of LPPCs = (1 − (A_1_)/A_2_) × 100%

where A_1_ is the proanthocyanidin standard equivalent in recovered LPPCs and A_2_ represents the proanthocyanidin standard equivalent in initial LPPCs.

#### 2.3.4. Determination of LOPCs in the Products

The p-dimethylaminocinnamaldehyde (DMAC) method was used for a determination as follows: prepare a 70% ethanol solution containing 12.5% concentrated hydrochloric acid, use the solution to dilute 0.05 g DMAC to 50 mL as a reaction solution; store in a 4 °C refrigerator protected from light; add 70 μL of sample solution to a 96-well plate; take 210 μL of DMAC reaction solution and add it quickly; react at 25 °C in the dark for 25 min; and measure the absorbance at a wavelength of 640 nm. The acidified ethanol solution was used to replace the DMAC reaction solution as the blank reagent group; the ultrapure water was used to replace the sample solution as the sample blank group [22]. The determination result was expressed with the proanthocyanidin standard equivalent per mg of LPPCs (μg GSP/mg DW).

The next step is to accurately prepare 1 mg/mL proanthocyanidin standard mother solution, and dilute the mother solution to obtain 0.01 mg/mL, 0.02 mg/mL, 0.04 mg/mL, 0.06 mg/mL, 0.08 mg/mL, and 0.1 mg/mL proanthocyanidin standard solution, according to the above methods, and determine and draw the concentration–absorbance standard curve, the equation is: y = 6.3964x – 0.0466, R² = 0.997.

#### 2.3.5. Determination of the Total Phenol Content (TPC) in the Products

A volume of 1 mL of a sample solution was added to 5 mL of Folin–Ciocalteu phenol reagent (10%). After mixing well and keeping standing for 5 min, the Na_2_CO_3_ solution (7.5%) was added. After homogeneous mixing, it was sealed and protected from light at room temperature for 60 min. The OD_765nm_ value was measured. In the blank control group, the sample solution was replaced with methanol. The determination result is expressed with the gallic acid equivalent (GAE) per mg of LPPCs (μg GAE/mg DW). A standard curve was obtained as follows. An amount of 1.0 mg of gallic acid standard substance was dissolved in methanol and diluted to a set volume of 10 mL. A mother solution with a concentration of 0.1 mg/mL was prepared and diluted with methanol. Then, various gallic acid standard solutions (10 μg/mL, 20 μg/mL, 30 μg/mL, 40 μg/mL, and 50 μg/mL) were made respectively [23]. After measuring the OD_765nm_ values of reaction solutions by using the above method, the linear relationship between the concentration of gallic acid and the absorbance value was acquired as follows: y = 0.0067x + 0.00510, R² = 0.9988.

### 2.4. HPLC-QTOF-MS/MS Analysis of the Products

In order to compare the changes in the products before and after fermentation, the fermentation time was set to be 12 h and 48 h when other factors remained unchanged. The LPPCs culture solution (48 h) without inoculation and the *Lactobacillus plantarum* fermentation broth (48 h) without containing LPPCs received the same treatment and were used as control-1 and control-2, respectively. Finally, the methanol solution of each sample was filtered by using a 0.22 μm filtration membrane.

The conditions of liquid chromatography were set as follows. The mobile phase included 0.1% formic acid water (A) and methanol (B). The chromatographic column referred to ZORBAX RPHD Eclipse Plus (C18, 2.1 mm × 100 mm, 1.8 μm). The injection volume was 10 μL, the flow rate is 0.4 mL/min, and the column temperature was set at 30 °C. The gradient elution program was as follows: 0–min, 10% B; 5–10 min, 10–30% B; 10–15 min, 30–50% B; 15–20 min, 50% B; 20–21 min, 50–10% B; 21–25 min, 10% B.

The conditions of mass spectrometry were specified as follows: Triple-TOF^TM^ 5600+ triple quadrupole time-of-flight mass spectrometer (AB SCIEX, USA); electrospray ion source, positive and negative ion modes; 30 psi for curtain gas, 50 psi for atomizing gas, 50 psi for desolvation gas, and 500 °C for desolvation gas temperature. The atomization potential was 4500 V and declustering potential is 100 V for the positive ion mode. The atomization potential was −4500 V and declustering potential was −100 V for the negative ion mode. The TOF MS scan range is 100–1000 *m*/*z*, and the TOF MS/MS scan range was 50–1000 *m*/*z*. The DBS mode (dynamic background subtraction) was adopted for scanning, and the IDA mode was utilized for acquisition. TOF-MS (250 ms) triggered 4 TOF-MS/MS (100 ms). The data were calibrated by utilizing the DuoSpray^TM^ ion source and Automated Calibrant Delivery System (SCIEX, Concord, Canada).

### 2.5. Determination of the In Vitro Antioxidative Activity

Details about measuring the antioxidative capacity of liposomes, DPPH scavenging activity, ABTS^+^ scavenging activity, and oxygen radical absorbance capacity (ORAC) were in Method 1 under the Supplementary Document of this manuscript.

### 2.6. Data Processing

The mass spectrometric data were processed and analyzed by using the SCIEX PeakView™ software and the online database Chemspider. The activity-related experimental data were processed and analyzed by utilizing Excel, GraphPadPrism 7 and SPSS 23 parallel groups were established for each experiment, and *p*-values less than 0.05 were considered significant differences.

## 3. Results and Discussion

### 3.1. Results of Screening Five Lactobacillus Species

#### 3.1.1. The Growth Curves of Lactobacilli during Transformation

**Figure 1:**
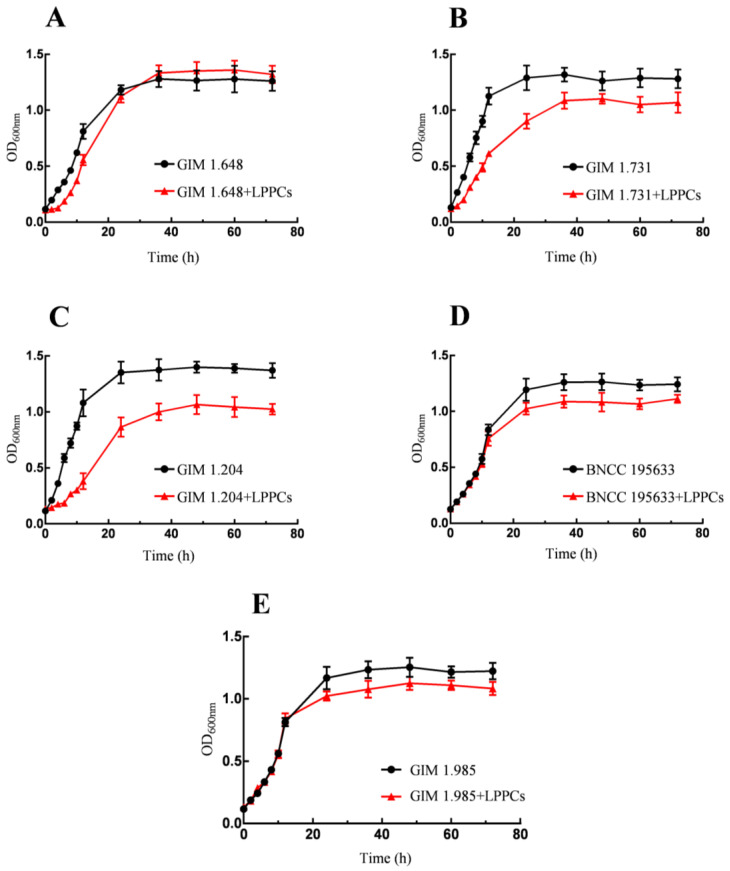
Growth curve of five Lactobacillus species during bioconversion on LPPCs ((**A**): *Lactobacillus plantarum*; (**B**): *Lactobacillus fermentum*; (**C**): *Lactobacillus acidophilus*; (**D**): *Lactobacillus casei*; (**E**): *Lactobacillus paracasei*).

As shown in Figure 1, after LPPCs were added to the culture medium, the growth of *Lactobacilli* was affected to various degrees, mainly represented by the changes in the lag phase and logarithmic phase in the growth cycle. According to the Figure 1B,C, after the addition of LPPCs, there were longer lag phases and significantly shorter logarithmic stages for *Lactobacillus fermentum* and *Lactobacillus acidophilus*, indicating a greater inhibitory effect of LPPCs on these two bacteria. According to the Figure 1D,E, the growth trends of *Lactobacillus casei* and *Lactobacillus paracasei* were similar. In the first fermentation period of 12 h, LPPCs had little influence on them. However, in a later period, the maximum OD values of both species were lower than those of their respective control group. With regard to the growth curve of *Lactobacillus plantarum* under the influence of LPPCs in Figure 1A, its lag phase was extended. During the period between 12 h and 24 h, there was a significant rise in the OD value, and the maximum OD value was basically the same as that of the control group.

#### 3.1.2. Changes in pH during Fermentation

A change in pH is one of the most direct indicators in observing the fermentation process of *Lactobacilli* [24]. The pH changes in the process of transformation of LPPCs by using five *Lactobacillus* species are shown in Table 1. When each strain fermented in a medium without LPPCs, the pH value was finally stable at 3.5–3.8. In the case of mediums with the same initial pH value, there was a smaller decreasing range for the pH value and an increase in the minimum pH value in terms of the four strains except for *Lactobacillus plantarum* during fermentation in mediums containing LPPCs, revealing that LPPCs affected the normal metabolic activities of bacteria to a certain degree and weakened their acid production capability. Among them, *Lactobacillus acidophilus* and *Lactobacillus fermentum* suffered the most significant influence. The acid production capability of *Lactobacillus plantarum* was affected by LPPCs to a low extent. In the later stage of fermentation, its pH value was 3.57 ± 0.05, which was not significantly different from that of the control group. It could overcome the affection of LPPCs to a certain extent and presented a good growth trend. This is probably due to the generation of some proanthocyanidin monomers after depolymerization of LPPCs during the period of 12–24 h, which accelerated the growth of *Lactobacillus plantarum*.

#### 3.1.3. Transformation Rates of LPPCs Based on Five Lactobacillus Species

**Figure 2:**
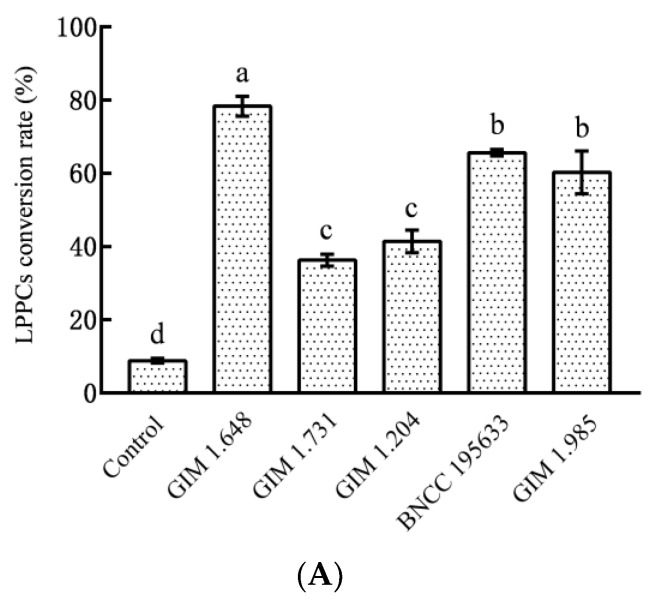

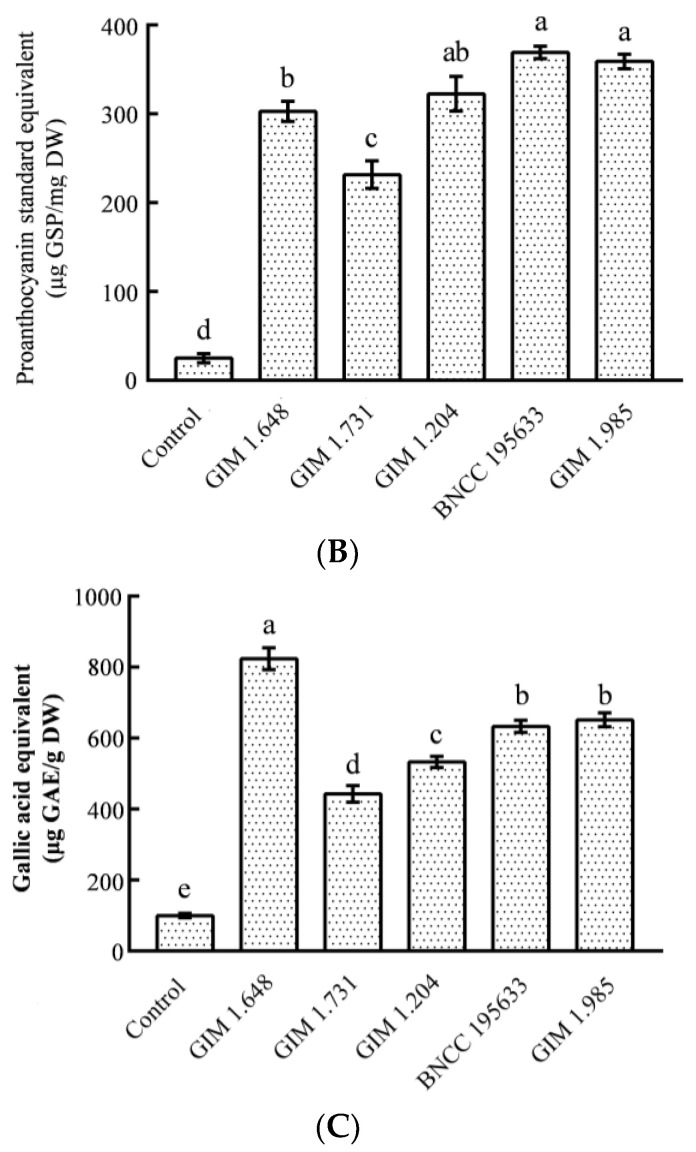
(**A**) Biotransformation rate of LPPCs by five *Lactobacillus*; (**B**) Oligomeric proanthocyanidins content in biotransformation products of LPPCs; (**C**) Total phenolics content in biotransformation products of LPPCs; Different letters indicate significant data differences (*p* < 0.05).

According to Figure 2A, the five *Lactobacillus* species transformed LPPCs to different degrees. *Lactobacillus plantarum* had the strongest capability to transform LPPCs, and after 72-h fermentation, the transformation rate reached 78.36 ± 2.71%. In terms of *Lactobacillus casei* and *Lactobacillus paracasei*, there was no significant difference between the transformation rates of LPPCs, and the transformation rates were 65.65 ± 0.87% and 60.27 ± 5.83%, respectively. *Lactobacillus fermentum* and *Lactobacillus acidophilus* were even weaker in transforming LPPCs, and the transformation rates were 36.25 ± 1.60% and 41.43 ± 3.10%, respectively. The above analysis revealed a higher degree of transformation of LPPCs under the influence of *Lactobacillus plantarum*, compared with the other four bacteria. Its transformation effect needs to be further discussed after considering the products from transformation.

#### 3.1.4. Analysis of the LOPCs Content in the Products

*Lactobacilli*, by means of carbon sources such as glucose, ferment and produce organic acids dominated by lactic acid, thus making the fermentation system faintly acidic. H^+^ catalyzes the cleavage of internal flavan bonds between proanthocyanidin monomers, which is a key process for the depolymerization of proanthocyanidins [25,26]. After 72 h fermentation, the products from transformation of LPPCs under the influence of various strains were acquired by using ethyl acetate to extract the fermentation broth. Among the proanthocyanidins, only flavanol monomers, proanthocyanidin oligomers, and other small-molecule structures can dissolve in ethyl acetate, while polymers with larger molecules fail to dissolve. Therefore, the detection of LOPCs in the products can help understand the conditions concerning the depolymerization of LPPCs into flavanol monomers or proanthocyanidin oligomers in the biotransformation system. As Figure 2B showed, the LOPCs contents detected in the products of LPPCs transformed by five *Lactobacillus* species were significantly (*p* < 0.05) higher than those in the control groups, and there was no remarkable difference between the LOPCs contents in the products from transformation based on the five *Lactobacillus* species. Specifically, the LOPCs content in the *Lactobacillus casei* group was the highest, reaching 369.16 ± 6.88 μg GSP/mg DW, which was not significantly different from those in the *Lactobacillus paracasei* group and *Lactobacillus acidophilus* group. The LOPCs content in the *Lactobacillus plantarum* group was 302.84 ± 11.46 μg GSP/mg DW. The LOPCs content in the *Lactobacillus fermentum* group was the lowest.

#### 3.1.5. Total Phenol Content (TPC) in the Products

Phenolic acid is one main category of dietary polyphenols. Relevant studies have indicated that after the fermentation by probiotics such as lactic acid bacteria, polyphenols could be transformed into small-molecule substances such as phenolic acids, leading to higher bioavailability and enhanced physiological activities such as radical ion scavenging activity [27,28]. As shown in Figure 2C, the TPC after transformation of LPPCs was significantly (*p* < 0.05) higher than those in the control groups. The *Lactobacillus plantarum* group had the highest TPC which reached 823.59 ± 30.92 μg GAE/g DW, followed by the *Lactobacillus paracasei* group and the *Lactobacillus casei* group (there was no significant difference between the TPCs in the two groups). The *Lactobacillus fermentum* group had the lowest TPC, only reaching 443.88 ± 23.38 μg GAE/g DW.

In summary, the findings concerning the growth and metabolism of five *Lactobacillus* species during the transformation process and their transformation effect on LPPCs confirm that *Lactobacillus plantarum* is the ideal strain for transformation of LPPCs. It will be used for further transformation-related experiments.

### 3.2. HPLC-QTOF-MS/MS Analysis of the Products

**Figure 3:**
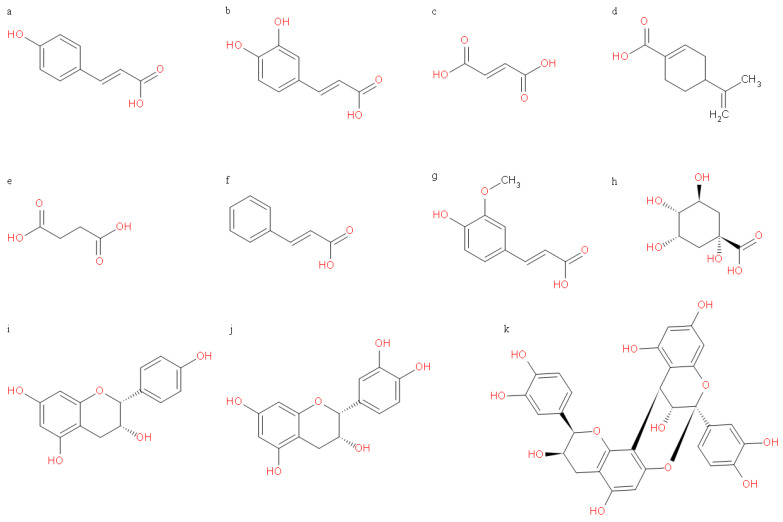
Chemical structures of detected compounds during or after biotransformation. ((**a**): 4-Hydroxycinnamic acid; (**b**): 3,4-Dihydroxybenzeneacrylic acid; (**c**): Fumaric acid; (**d**): Perillic acid; (**e**): Succinic acid; (**f**): Cinnamic acid; (**g**): 3-(4-Hydroxy-3-methoxyphenyl) propenoic acid; (**h**): Quinic acid; (**i**): Afzelechin; (**j**): Epicatchin; (**k**): Procyanidin A2).

Matching and detection of data were carried out based on databases. In combination with MS/MS, the information about structures such as main fragment ions of related substances in different literature and reports were analyzed and compared. Then, 11 substances were found from intermediate products or end-products, as shown in Figure 3. They were dominated by phenolic acids with different structures, including 4-hydroxycinnamic acid, 3,4-dihydroxybenzeneacrylic acid, fumaric acid, perillic acid, succinic acid, 3-(4-hydroxy-3-methoxyphenyl) propenoic acid, cinnamic acid, quinic acid, afzelechin, epicatechin, and procyanidin A2. Among them, afzelechin and epicatechin were intermediate products at the 12th hour during fermentation, which were not found in the end products. Epicatechin and proanthocyanidin A2 were identified in the uninoculated blank control groups, revealing the natural degradation of LPPCs at 37 °C. Fumaric acid and succinic acid, also discovered in the blank control groups without containing LPPCs, were preliminarily considered two metabolites of *Lactobacillus plantarum*, rather than the products from transformation of LPPCs. Therefore, seven products from LPPCs transformed by *Lactobacillus plantarum* were preliminarily identified.

According to the results from data analysis by HPLC-QTOF-MS in Table 2, the change in the response peak area could suggest that 4-hydroxycinnamic acid, 3,4-dihydroxy-cinnamic acid and proanthocyanidin A2 were major components in the products from transformation of LPPCs. Compared with the conditions after the 12-h fermentation, the response peak areas for 4-hydroxycinnamic acid and 3,4-dihydroxy-cinnamic acid expanded remarkably, while there was little change in the response peak area for proanthocyanidin A2. This possibly meant that *Lactobacillus plantarum* could transform LPPCs into phenolic acid structures dominated by 4-hydroxycinnamic acid and 3,4-dihydroxy-cinnamic acid, but it failed to degrade proanthocyanidin A2. Fernando Sanchez-Patán et al. also obtained the same results when using *Lactobacillus plantarum* to ferment proanthocyanidin A2. This may be correlated with the special bond type of A-type proanthocyanidins [29]. 4-Hydroxycinnamic acid and 3,4-dihydroxy-cinnamic acid are common and minor products from microbial degradation of proanthocyanidins. They have been detected in studies related to in vitro microbial fermentation of proanthocyanidins or in vivo digestion [30,31]. On the other hand, the main products such as phenylacetic acid and phenylpropionic acid series found in other related studies were not detected in current study. The most significant difference between the two kinds of substances was that the organic acid on the phenyl group of the former substance was alkene acid while that of the latter substance was alkane acid [30,32,33]. This may result from the difference in the composition of LPPCs in the samples.

Based on the mass spectrometric data and the characteristics of easy depolymerization of polymeric proanthocyanidins under acidic conditions, a primary speculation in terms of the paths for transforming LPPCs by *Lactobacillus plantarum* was indicated as follows. In the initial stage, *Lactobacillus plantarum* decreased the pH value of the fermentation system by acid production, promoting the depolymerization of LPPCs and the generation of monomers with flavan-3-ol structures. Then, the enzyme system catalyzed degradation and metabolism, producing an intermediate metabolite called 1-(3′,4′-dihydroxyphenyl)-3-(2″,4″,6″-trihydroxyphenyl) propan-2-ol. After further metabolism, intermediate products such as 5-(3′,4′-dihydroxyphenyl)-γ-valerolactone and 4-hydroxy-5-(3′, 4′-dihydroxyphenyl) valeric acid were produced. Subsequently, a series of decarboxylation, dehydroxylation and oxidation reactions facilitated the generation of phenolic acids with different hydroxylation patterns.

### 3.3. A study of the In Vitro Antioxidative Activity

In order to study the changes in the in vitro antioxidative activity of the products, samples including LPPCs, LOPCs, products from transformation, and main presumed components such as 4-hydroxycinnamic acid and 3,4-dihydroxy-cinnamic acid were used in subsequent experiments to compare the changes in antioxidative activity before and after transformation of LPPCs and analyze the effect and influence of 4-hydroxycinnamic acid and 3,4-dihydroxy-cinnamic acid in the change in activity.

#### 3.3.1. Antioxidative Capacity of Liposomes

Lecithin was used to prepare artificial liposomes. Copper acetate was added to trigger peroxidation reactions to simulate the oxidation of biological membranes. The anti-lipid peroxidations of different samples were compared by considering the concentration curves of CD-POV (the main primary product in the process of lipid peroxidation) and inhibition rates [34].

**Figure 4:**
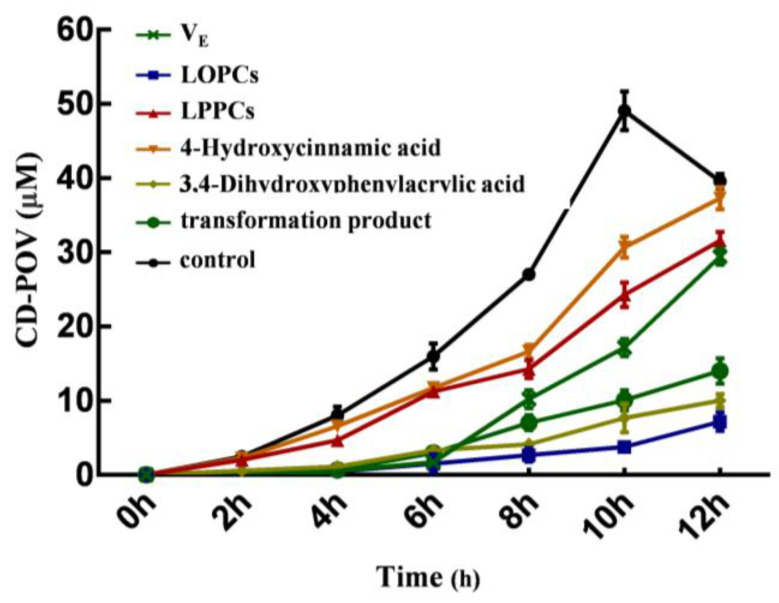
Antioxidant activity on liposome system before and after LPPCs biotransformation.

Within 12 h of the lipid peroxidation reaction, the concentration of CD-POV in the blank control group peaked at the 10th hour, while the concentration in other sample groups still showed an upward trend at the 12th hour (Figure 4), indicating that other sample groups took longer time to reach the peak than the blank control group. Furthermore, the concentrations of CD-POV in other sample groups were lower than that in the blank control group at the same time points, revealing that test samples inhibited lipid oxidation to various degrees. The inhibition rate was calculated according to the maximum concentration of each sample group within 12 h of reaction (Table 3). When the concentration of each sample was the same, the products from transformation had a greater inhibitory effect on lipid peroxidation than LPPCs and V_E_ but showed a slightly smaller inhibitory effect than LOPCs (its inhibition rate reached 71.42%). The inhibition rate of 4-hydroxycinnamic acid was only 24.03%, demonstrating that it was not the dominant substance leading to the anti-lipid peroxidation of the products.

#### 3.3.2. Determination of the DPPH Scavenging Activity, ABTS^+^ Scavenging Activity, and ORAC Capability

**Figure 5:**
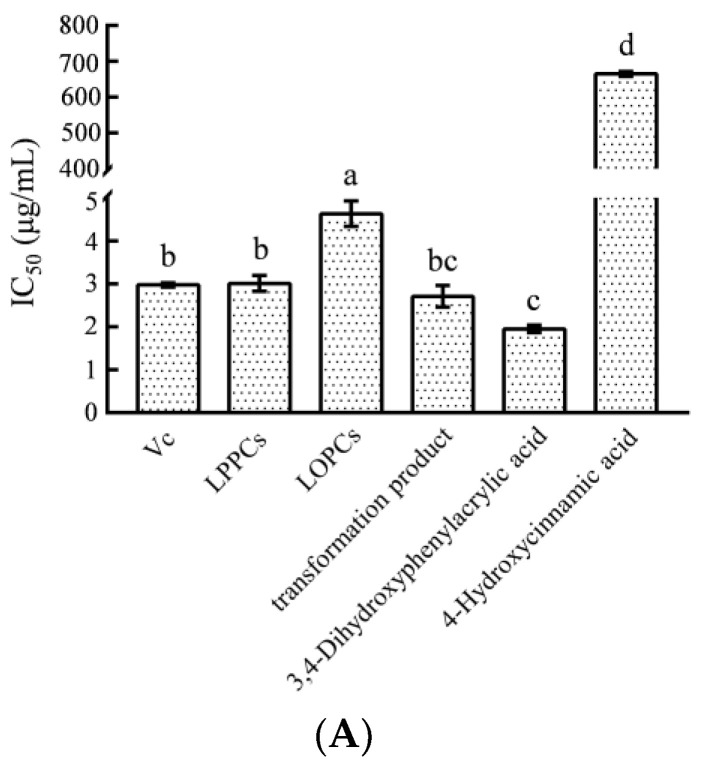

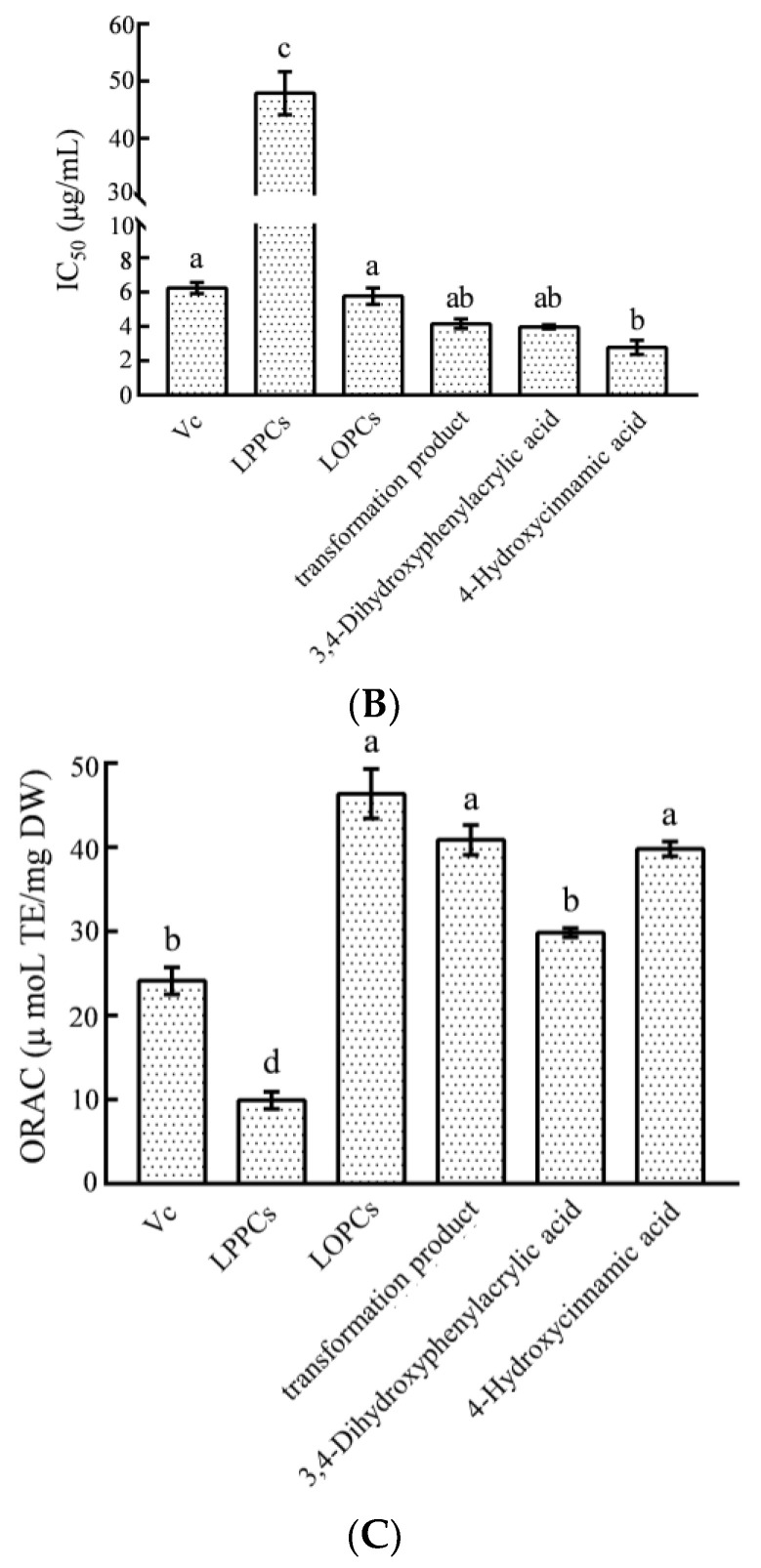
(**A**) Change in scavenging ability on DPPH·radical before and after LPPCs biotransformation; (**B**) Change in scavenging ability on ABTS^+^·radical before and after LPPCs biotransformation; (**C**) Change in ORAC value before and after LPPCs biotransformation; Different letters indicate significant data differences (*p* < 0.05).

The DPPH radical scavenging capability is commonly used for determining the in vitro antioxidative property, expressed by a clearance rate or IC_50_ value. A lower IC_50_ value, or a higher clearance rate in the case of the same concentration, means greater DPPH scavenging activity [35]. As shown in Figure 5A, the IC_50_ value for DPPH radical scavenging by the products from transformation was not significantly (*p* > 0.05) different from those in terms of LPPCs and V_C_, but was significantly lower than that for DPPH radical scavenging by LOPCs. According to IC_50_-based calculations, the DPPH scavenging activity of the products from transformation was 1.71 times that of LOPCs. The IC_50_ value of 4-hydroxycinnamic acid, as one of the major components of the products, was much higher than those of the other five substances, indicating that it had a weaker DPPH scavenging activity and did not impede the influence of the activity of the products. Hence, it is believed that the DPPH scavenging activity of the products depends on the synergy between different components [36].

ABTS^+^ radical scavenging capability is also commonly used for measuring the in vitro antioxidative activity [37]. According to Figure 5B, the IC_50_ value for ABTS^+^ radical scavenging by the products was significantly lower than that for ABTS^+^ radical scavenging by LPPCs. This revealed that the ABTS^+^ radical scavenging capability after fermentation and transformation of LPPCs was significantly improved (*p* < 0.05) and higher than those of the LOPCs group and the positive control VC group. The results also showed that 3,4-dihydroxy-cinnamic acid and 4-hydroxycinnamic acid had strong ABTS^+^ scavenging activities. According to the findings by further comparing the results of experiments concerning DPPH scavenging, the radical scavenging activity of the products from transformation was independent of a change in the solvent. Compared with the organic solvent system, the antioxidative activity in the aqueous phase system can reflect the conditions inside organisms to a greater extent. Therefore, the products from transformation have more excellent radical scavenging capability than LPPCs.

ORAC is a highly accurate and sensitive method for evaluating antioxidative activity. It is a general indicator recognized by the US Department of Agriculture and the Ministry of Health for evaluating the antioxidative capacity of foods. A larger ORAC value means a stronger oxygen radical absorbance capacity and greater antioxidative activity [36,38]. As shown in Figure 5C, after fermentation and transformation by *Lactobacillus plantarum*, the ORAC value of the products from transformation was 40.88 ± 1.78 μmol TE/mg DW, which was 4.13 times that of LPPCs and significantly higher than that of the positive control V_C_ group. Moreover, the ORAC value of the products from transformation was significantly (*p* < 0.05) greater than that of 4-hydroxycinnamic acid and close to that of 3,4-dihydroxy-cinnamic acid. This can be considered that the improvement in oxygen radical absorbance capacity after fermentation and transformation of LPPCs results from the increased number of phenolic acids and the synergy between them.

## 4. Conclusions

This study took litchi pericarp as a research object and selected a proper strain for transforming LPPCs from five common *Lactobacillus* species according to the growth conditions and transformation effect of bacteria. Then, the products from transformation were analyzed to speculate on their transformation paths and explore the antioxidative activity of the products. The results showed that LPPCs affected the growth and metabolism of the five *Lactobacillus* species to various degrees. Specifically, *Lactobacillus plantarum* was the most tolerant to LPPCs, and the rate of transforming LPPCs by it was the highest, reaching 78.36 ± 2.71%. The total phenolic content in the products was significant and reached 823.59 ± 30.92 μg GAE/g DW. Hence, it is the most suitable strain for the fermentation and transformation of LPPCs. By adopting HPLC-QTOF-MS/MS, seven kinds of substances were identified from the products, among which 4-hydroxycinnamic acid, 3,4-dihydroxy-cinnamic acid and proanthocyanidin A2 were considered major substances in the products. Moreover, the findings reveal that the products transformed by *Lactobacillus plantarum* show much higher in vitro antioxidative activity, effectively delay lipid peroxidation, and possess good ABTS^+^ (IC_50_: 4.16 ± 0.28 μg/mL) and DPPH (IC_50_: 2.86 ± 0.19 μg/mL) radical scavenging capabilities and ORAC capability (40.88 ± 1.78 μmol TE/mg DW). *Lactobacilli* were selected to transform polymeric proanthocyanidins, generating products with highly antioxidative activity. This study suggests the potential utilization of by-products from litchi processing and polymeric proanthocyanidins.

## Figures and Tables

**Table 1:** Change in pH during bioconversion on LPPCs by five *Lactobacillus.*

Times		0 h	24 h	48 h	72 h
*Lactobacillus plantarum*	LPPCs	5.80 ± 0.01 ^a^	3.79 ± 0.04 ^b^	3.60 ± 0.06 ^a^	3.57 ± 0.05 ^a^
	Control	5.81 ± 0.01 ^a^	3.65 ± 0.01 ^a^	3.58 ± 0.01 ^a^	3.54 ± 0.01 ^a^
*Lactobacillus fermentum*	LPPCs	5.80 ± 0.01 ^a^	4.19 ± 0.05 ^d^	4.20 ± 0.01 ^c^	4.21 ± 0.03 ^d^
	Control	5.80 ± 0.00 ^a^	3.84 ± 0.01 ^bc^	3.80 ± 0.01 ^b^	3.77 ± 0.01 ^bc^
*Lactobacillus acidophilus*	LPPCs	5.80 ± 0.01 ^a^	4.15 ± 0.03 ^d^	4.11 ± 0.03 ^c^	4.12 ± 0.01 ^d^
	Control	5.81 ± 0.01 ^a^	3.75 ± 0.01 ^b^	3.69 ± 0.01 ^ab^	3.68 ± 0.01 ^b^
*Lactobacillus casei*	LPPCs	5.81 ± 0.01 ^a^	3.93 ± 0.02 ^c^	3.87 ± 0.04 ^b^	3.84 ± 0.04 ^c^
	Control	5.81 ± 0.00 ^a^	3.68 ± 0.01 ^ab^	3.61 ± 0.01 ^a^	3.60 ± 0.01 ^ab^
*Lactobacillus paracasei*	LPPCs	5.81 ± 0.01 ^a^	3.92 ± 0.03 ^c^	3.88 ± 0.04 ^b^	3.89 ± 0.02 ^c^
	Control	5.81 ± 0.01 ^a^	3.70 ± 0.01 ^ab^	3.65 ± 0.01 ^a^	3.63 ± 0.01 ^ab^

The data indicated with various letters at the same time point after the Tukey-test test indicate a significant difference (*p* < 0.05). Results are expressed as mean ± standard deviation (*n* = 3).

**Table 2:** HPLC-QTOF-MS data of the detected compounds during or after biotransformation.

NO.	Retention Time (min)	Molecular Formula	Preliminary Identification of Substances	Relative Molecular Mass (g/mol)	[M^+^H] ^+^ Measured Value m/z	Major Fragment Ion *m*/*z*	Response Peak Area			
							Control-1 48 h	Control-2 48 h	12 h	48 h
1	1.57	C_4_H_6_O_4_	Succinic acid	118.09	119.04	55/59/73/101	NF	<10^4^	1.0 × 10^4^	2.2 × 10^4^
2	1.73	C_4_H_4_O_4_	Fumaric acid	116.07	117.02	73/99	NF	<10^4^	1.2 × 10^4^	2.0 × 10^4^
3	10.05	C_9_H_8_O_4_	**3,4-Dihydroxybenzeneacrylic acid**	180.15	181.05	72/119/134/135/163	NF	NF	1.5 × 10^4^	7.3 × 10^5^
4	11.14	C_15_H_14_O_6_	Epicatechin	290.27	291.10	109/137/179/185/245	3.1 × 10^4^	NF	8.2 × 10^5^	NF
5	16.67	C_7_H_12_O_6_	Quinic acid	192.17	193.07	92/104/131/148/175	NF	NF	1.3 × 10^4^	3.1 × 10^4^
6	17.32	C_30_H_24_O_12_	Procyanidin A2	576.51	577.17	287/291/299/311/407/411/425/437	2.6 × 10^4^	NF	3.6 × 10^5^	5.4 × 10^5^
7	17.93	C_10_H_14_O_2_	Perillic acid	166.22	167.10	74/103/119/147/149	NF	NF	5.1 × 10^4^	5.5 × 10^4^
8	18.05	C_9_H_8_O_3_	4-Hydroxycinnamic acid	164.16	165.06	72/91/101/103/117/119/147	NF	NF	6.6 × 10^4^	9.3 × 10^5^
9	18.63	C_15_H_14_O_5_	Afzelechin	274.27	275.10	109/127/147/169	NF	NF	3.0 × 10^5^	NF
10	21.07	C_10_H_10_O_4_	3-(4-Hydroxy-3-methoxyphen-yl) propenoic acid	194.19	195.07	121/136/151/177	NF	NF	NF	1.7 × 10^4^
11	23.94	C_9_H_8_O_2_	Cinnamic acid	148.17	149.06	39/43/57/59/75/83/87	NF	NF	NF	8.1 × 10^4^

“NF” means Not found.

**Table 3:** The inhibition rate of different ingredients on CD-POV.

Substance	V_E_	LOPCs	LPPCs	Transformation Product	4-Hydroxycinnamic Acid	3,4-Dihydroxyphenylacrylic Acid	Control
Maximum concentration (μM)	29.43 ± 1.11	31.58 ± 1.25	7.16 ± 1.17	14.02 ± 17.1	37.27 ± 1.50	10.00 ± 0.92	49.06 ± 2.61
Inhibition rate (%)	40.01	85.41	35.63	71.42	24.03	79.62	0

The maximum concentration is expressed as the mean ± SD, and the inhibition rate is calculated as the mean of the maximum concentration.

## Data Availability

Data is contained within the article or Supplementary Materials.

## Supplementary Materials

The following supporting information can be downloaded at: https://www.mdpi.com/article/10.3390/foods12122384/s1, References [39,40,41,42] are cited in the Supplementary Materials (Supporting method information).
