# Supplementary material for: Boosting the Antioxidant Potential of Polymeric Proanthocyanidins in Litchi (Litchi chinensis Sonn.) Pericarp via Biotransformation of Utilizing Lactobacillus Plantarum
Source: Foods. 2023 Jun 15;12(12):2384. doi: 10.3390/foods12122384 (PMC10297231; doi:10.3390/foods12122384)
Supplement: Supplementary file 1 [file foods-12-02384-s001.zip › foods-2429242-supplementary.pdf]

**Supporting information**

## 1. *In vitro* antioxidant activity assay

### 1.1. Liposome Antioxidant Capacity Determination

Accurately weigh lecithin and dissolve it in 10 mM PBS buffer (pH 7.4, 150 mM NaCl), and sonicate in an ice-water bath for 30 min to prepare artificial liposomes with a concentration of 1.0 mg/mL. Dissolve the test sample in methanol to prepare a sample solution with a concentration of 0.5 mg/mL, take 0.20 mL and dissolve it in 19.8 mL of artificial liposome, add 20 μL of copper acetate, and place it in a 37°C water bath shaking shaker protected from light, start the lipid oxidation reaction, react for 12 h, and take samples every 2 h to determine the concentration of conjugated diene hydroperoxide (CD-POV). The sample solution was replaced by methanol as a blank control, and three parallel groups were set up for each experiment [39].

Determination of conjugated diene hydroperoxide (CD-POV): Dilute 1.0 mL liposome oxidation solution 5 times with methanol, dilute 1.0 mL PBS buffer solution 5 times with methanol as a reference, and measure it at 234nm. The absorbance of the measured value minus the absorbance of 0h is the absorbance of the conjugated diene hydroperoxide. With the molar extinction coefficient ε=26000 M-1cm-1, the concentration of CD-POV in the liposome oxidation solution at each time point was calculated according to the Lambert-Beer law, and the lipid oxidation inhibition rate of each sample was calculated according to the maximum concentration:

$$\text{Transformation rate of LPPCs} = (1 - B_1/B_2) \times 100\%$$

Where $B_1$ is the maximum concentration of CD-POV in the experimental group, $B_2$ represents the maximum concentration of CD-POV in the blank group.

### 1.2. Clearance of DPPH activity assay

Accurately weigh 2,2-biphenyl-1-picrylhydrazine (DPPH·) and dissolve it in methanol to prepare a DPPH solution with a concentration of 0.5 mM; mL, 10 μg/mL, 50 μg/mL, 100

μg/mL, 500 μg/mL, 1000 μg/mL methanol solution, take 100 μL DPPH solution in a 96-well plate, quickly add 100 μL sample solution, keep at room temperature in the dark After 30 min of reaction, the OD517nm was measured. The half inhibitory concentration IC50 was calculated. The sample solution was replaced by methanol as the blank control; the DPPH solution was replaced by methanol as the blank reagent control. Three parallel groups were set up for each experiment [40].

**1.3. Clearance $ABTS^+$ activity assay**

Accurately weigh ABTS and dissolve it in 10 mM PBS buffer solution (pH 7.4, 150 mM NaCl). Prepare an ABTS solution with a concentration of 2.5 mM, add an appropriate amount of manganese dioxide, react overnight at 30°C, pass through a 0.45 μm filter membrane, dilute a certain number of times so that the OD734nm value is about 0.6-0.8, and store it in a refrigerator at 4°C in the dark for future use; Prepare the measurement samples into PBS solutions with concentrations of 1 μg/mL, 5 μg/mL, 10 μg/mL, 50 μg/mL, 100 μg/mL, 500 μg/mL, and 1000 μg/mL, and take 180 μL of ABTS solution Add 20 μL of sample solution quickly to a 96-well plate, react in the dark for 10 min, measure OD734nm, and calculate the half inhibitory concentration IC50. PBS buffer was used instead of sample solution as blank control; PBS buffer was used instead of ABTS solution as blank reagent control, and three parallel groups were set up for each experiment [41].

**1.4. Oxidative radical absorbance capacity (ORAC) assay**

Method references with modifications [42], accurately prepare 70 nM sodium fluorescein solution and 40 mM 2,2-azobis (2-methylpropylimidium) dihydrochloride (AAPH·) solution with 10 mM PBS buffer (pH 7.4), and Store in the dark Store in a refrigerator at 4°C for later use; prepare Trolox with PBS buffer solution at a concentration of 20 μM, 40 μM, 60 μM, 80 μM and 100 μM; prepare the assay sample into a 100 μg/mL PBS solution. Add 20 μL of sample solution and 120 μL of sodium fluorescein solution to a 96-well black plate, place it in a preheated instrument and incubate for 15 min, then quickly

add 60 μL of AAPH solution to start the reaction, measure the fluorescence value every 1 min, and continue 120 min. PBS buffer was used instead of AAPH solution as a control for natural fluorescence quenching (AAPH-), and PBS buffer was used instead of sample solution as a blank control (AAPH+). The absolute fluorescence intensity at each time point of the sample group and the AAPH group was compared to obtain the relative fluorescence intensity $f_n$, and the area of the fluorescence quenching curve (AUC) was calculated using the approximate integral method with $f_n$, the formula is as follows:

$$\mathrm{AUC} = \sum_{n=1}^{120} \frac{1}{2}\left(f_{n-1} + f_n\right)$$

Then calculate net AUC=AUC $_{sample}$-AUC$_{AAPH+}$, and the ORAC value of each sample is expressed in Trolox (μmol TE/mg DW) equivalent.
